# The Management of Spinal and Epidural Anesthesia-Related Hypotension in the United States During Cesarean Childbirth

**DOI:** 10.7759/cureus.56340

**Published:** 2024-03-17

**Authors:** Harshita Nadella, Aditi Islam, Emily A Ina, Dianna Levin, Toni Bacoat-Jones

**Affiliations:** 1 Rheumatology and Immunology, Nova Southeastern University Dr. Kiran C. Patel College of Osteopathic Medicine, Davie, USA; 2 Obstetrics and Gynecology, Nova Southeastern University Dr. Kiran C. Patel College of Osteopathic Medicine, Fort Lauderdale, USA; 3 Osteopathic Medicine, Nova Southeastern University Dr. Kiran C. Patel College of Osteopathic Medicine, Fort Lauderdale, USA; 4 Osteopathic Medicine, Nova Southeastern University Dr. Kiran C. Patel College of Osteopathic Medicine, Davie, USA; 5 Osteopathic Medicine, Nova Southeastern University Dr. Kiran C. Patel College of Osteopathic Medicine, Clearwater, USA

**Keywords:** c-section, obstetric and gynecology anesthesia, post spinal hypotension, spinal-epidural anesthesia, obstetric epidural

## Abstract

This study delves into the prevalence of spinal anesthesia-induced hypotension during cesarean (c-section) childbirth, focusing on existing treatments and their efficacy. Currently, neuraxial analgesia is the most efficient method for alleviating pain during c-sections, but its major side effect, hypotension, necessitates a thorough understanding of the available treatment options. A scoping review was conducted using PubMed and Rayyan, with inclusion criteria being English peer-reviewed articles from the last five years, involving nulligravida/primigravida women under 35 years old in the United States.

The research reveals various treatments to mitigate spinal anesthesia-induced hypotension. Norepinephrine and epinephrine have demonstrated effectiveness in maintaining blood pressure while reducing adverse maternal outcomes following delivery. When comparing fixed-rate infusions of norepinephrine to phenylephrine, norepinephrine demonstrated lower rates of bradycardia (*p*=0.004), thereby reducing the necessity for bolus atropine rescue (*p*=0.01). Furthermore, the use of colloid solutions during c-sections significantly decreased the incidence of hypotension when compared to crystalloid solutions (*p*<0.00001). Non-pharmacological methods, such as lower extremity wrapping and elevation, exhibited higher systolic and diastolic blood pressures, along with higher usage of ephedrine when compared to control groups.

Pharmacological treatments proved more effective than non-pharmacological interventions in preventing maternal hypotension during c-sections. Notably, colloid preloading emerged as the most effective approach, helping to maintain maternal blood pressure, cardiac output, and heart rate while also minimizing the amount of ephedrine required and reducing anesthesia-related adverse effects. However, the study suggests the need for further investigations to determine the optimal dosage for colloid preloading. This research provides valuable insights into enhancing maternal well-being during c-sections by addressing the issue of neuraxial anesthesia-induced hypotension.

## Introduction and background

The modern era of childbirth analgesia commenced in 1847 when Dr. James Young Simpson administered ether to a woman in childbirth. Later in the same year, chloroform was introduced for the same purpose. Between 1900 and 1930, publications for descriptions of spinal, lumbar, caudal epidural, paravertebral, and pudendal nerve blocks for obstetrics were first recognized. The practice of neuraxial analgesia emerged in the mid-20th century when the first report of continuous caudal analgesia for childbirth was published in 1943 by Hingson and Edwards [1]. Presently, neuraxial labor analgesia stands as the most comprehensive and effective method for pain relief during childbirth and is the only method that offers complete analgesia without inducing maternal or fetal sedation [1].

The most common neuraxial analgesia types used in U.S. hospitals are continuous lumbar epidural and combined spinal-epidural analgesia. There are multiple side effects such as hypotension, perineal tears, issues with breastfeeding, and abnormal fetal head position during delivery [2]. There have been a number of advancements and new technologies utilized in labor and delivery. Despite this, in the U.S., the maternal mortality rate in 2020 was 23.8 deaths per 100,000 births which has increased since 2019 and is a 26.6% increase since 2000 [3]. This demonstrates the vital need to assess the current risks of neuraxial anesthesia to maternal health.

Hypotension is a frequently encountered complication caused by neuraxial anesthesia, which may adversely impact the parturient's condition [4]. The main physiological ways that hypotension can manifest are through a reduction in cardiac output, systemic venous resistance, and preload. Studies showed in severe cases, hypotension may ultimately result in hypoxia, acidosis, fetal distress, and reduced Apgar scores. Factors contributing to hypotension include increased blood loss, heightened parasympathetic drive, or decreased sympathetic tone. Studies also showed that there are a multitude of possible treatments and preventions of anesthesia-induced hypotension. 

One simple approach to managing blood pressure is through the administration of intravenous (IV) fluids. In addition to IV fluids, many pharmacological treatments exist such as the use of phenylephrine, which activates alpha-1 receptors, resulting in vasoconstriction. Non-selective adrenergic agonists such as ephedrine can also be used in the management of maternal hypotension. However, while ephedrine has been effective in limiting uteroplacental vasoconstriction, it poses a risk to fetal health by potentially causing umbilical cord acidosis [4]. Non-pharmacological methods encompass leg wrapping, the use of colloid solutions and placing the patient in the Trendelenburg position [4].

Current researchers have investigated the administration of colloid preload during neuraxial (spinal) anesthesia to see how well it can reduce the risk of hypotension during cesarean sections [5]. Gong et al. found after analyzing 831 patients in a total of nine studies, that there were no significant differences in hypotension between those who received colloid preload and the control group [5]. The researchers also found that hypotension occurring during spinal anesthesia is an important factor in intra-operative nausea and vomiting due to gut ischemia and serotonin release. However, there were no significant differences in hypotension levels compared to the control group when colloid was administered [5]. Additionally, when analyzing volume therapy, a rapid crystalloid co-loading is more effective in reducing the incidence of hypotension than a crystalloid preload [6]. Nonetheless, as Massoth et al. state, the choice of vasopressor to reduce the incidence of hypotension is multifactorial and depends on the state of the mother and fetus [6]. 

Studies have also been conducted to analyze the comparative efficacy of phenylephrine versus epinephrine to try and combat hypotension through alpha-1 receptor agonists [7]. Multiple studies have tried to determine the optimal dosing, time to administer, and the most effective agonist. This study aims to elucidate the most efficacious improvements to treating anesthesia-induced hypotension, compare existing solutions, and highlight their strengths and weaknesses.

Methods

To get a basic understanding of the existing literature, a broad search using the Joanna Briggs Institute (JBI) methodology for a scoping review was utilized via the PubMed database with keywords such as spinal anesthesia, hypotension, and maternal adverse effects. The JBI methodology uses an evidence-based approach that considers the feasibility, appropriateness, meaningfulness, and effectiveness (FAME) of each article. The base search strategy was constructed by analyzing key terms in Rayyan and from relevant articles in PMID, Turkish Journal of Medical Sciences, Chinese Medical Journal, JAMA Network Open, Anesthesiology, PMCID, and BMC Pregnancy Childbirth. Researchers then limited articles to those written after January 1st, 2018, and this base search was adapted to each database. To meet inclusion criteria, articles had to be peer-reviewed, written in English, and based on research studies taking place at any time within the last five years. Other criteria included women under 35 years old with no pre-existing comorbidities, residing and receiving health care in the United States, and c-section births. The researchers chose to limit the analysis to studies in the U.S. due to various hospital protocols that might impact the standard of care amongst patients worldwide. Additionally, the research included criteria limitations to nulligravida and primigravida, given the increased chance of complications with multigravida.

Researchers performed the initial search on September 9th, 2022, and used the Population, Concept, and Context (PCC) method to establish the population as healthy, full-term mothers. The concept involves the post-delivery anesthetic protocols in a review of different modalities used to prevent hypotension in mothers post neuraxial (epidural/spinal) anesthesia-induced maternal hypotension. Based on these and prior searches that produced a multitude of search results, there was a need to narrow the inclusion criteria due to excess data yielded from the existing criteria. Therefore, the researchers converged apparent themes within the research and decided to investigate “What effect, if any, does neuraxial analgesia (epidural and spinal anesthesia) have on the maternal outcome of hypotension after delivery?” For the initial search, electronic databases such as PubMed, Anesthesia BMC, and other databases within Embase. Researchers then conducted a second search using the refined search terms "anesthesia, childbirth, and maternal," in addition, the search was limited to 2018-2022, clinical articles/trials, comparative effectiveness/study, controlled study, double-blind procedure, major clinical study, meta-analysis, observational study, prospective study, randomized controlled trial, or retrospective study which yielded 154 article results. Two duplicates were excluded, leading to 152 articles. Researchers then excluded five articles that could not be accessed, leading to 147 articles. From there, the researchers were able to eliminate 140 resources due to the following reasons - 22 studies had participants with pre-existing conditions/comorbidities, 27 studies were performed outside of the United States, 29 studies discussed hypotension during or before pregnancy and were not specific to hypotension induced by anesthesia, 37 resources were the wrong type of study (not looking for drug effects, not looking for the treatment of hypotension but rather what adverse effects resulted from various anesthetics), 19 studies were not specific to the population of interest, and six studies had statistically insignificant findings with p values larger than 0.05. This left researchers in our group with seven studies to review. The reviewers then discussed each full-text article that was being considered for inclusion. When a disagreement occurred, a third independent reviewer helped to discuss and resolve the conflict.

Two authors then conducted the third research stage independently using Rayyan to organize results. The search strategy was reviewed by a librarian using the PRESS checklist. The authors consulted with a physician to ensure that the refined search question was relevant to clinical obstetrics and that the question was correctly phrased. In this review, researchers included studies that incorporated management of the hypotensive effects of epidural and spinal anesthesia on the maternal population post-delivery, published in English and dated after January 1st, 2018. Two authors also created the data-charting form to determine which variables to exclude versus include amongst the selected sources of evidence. The two reviewers independently charted the data and later collaborated to review the results. The researchers updated our data-charting form (Preferred Reporting Items for Systematic Reviews and Meta-Analyses, PRISMA) with findings throughout the process. Data was extracted on article characteristics, types, and dosage of each treatment for maternal hypotension post-spinal anesthesia and the results of each treatment on maternal hypotension. The studies were grouped by the medication protocols for post-spinal anesthesia-induced maternal hypotension. Researchers used a table format to organize the summaries and analyses of the chosen articles. Next, the population, study design, measures, and findings of the chosen articles were recorded in the tables for future reference.

**Figure 1:**
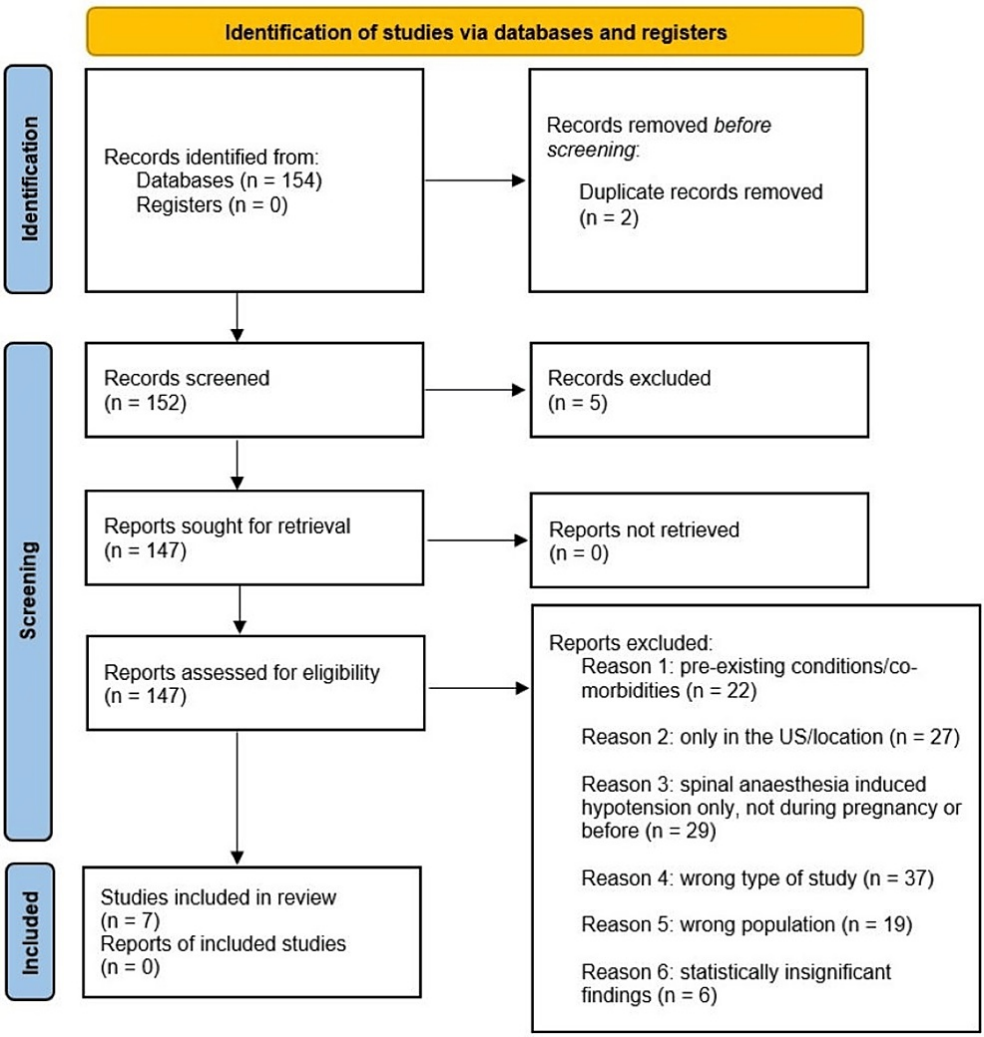
Preferred Reporting Items for Systematic Reviews and Meta-Analyses (PRISMA) Flow Diagram

Figure 1 shows the PRISMA flow diagram to demonstrate the search methods utilized by the research team. Within the seven studies of review, researchers grouped findings based on treatment modality, including - phenylephrine vs norepinephrine, other pharmacological treatments such as colloid vs crystalloid and methoxamine treatment, and lastly, non-pharmacological therapies such as leg wrapping. 

## Review

Results

Upon review of the selected articles in this study, researchers found that there were three main categories for which the management of hypotension induced by neuraxial analgesia (spinal and epidural)-related anesthesia could be grouped: common pharmacological treatments, additional pharmacological treatments, and non-pharmacological treatments. Common pharmacological treatments included epinephrine, norepinephrine phenylephrine, and body mass index (BMI) management. Additional pharmacological treatments for neuraxial analgesia (spinal and epidural anesthesia)-induced hypotension include colloid and crystalloid loading as well as prophylactic methoxamine infusions, while nonpharmacological treatments include leg wrapping.

Epinephrine vs Phenylephrine

Phenylephrine has been the vasopressor of choice in spinal anesthesia, even though it has resulted in bradycardia and decreased cardiac output. Goel et al. have sought to evaluate the effects of phenylephrine compared to norepinephrine infusion on maternal hemodynamics, including hypotension, intraoperative nausea/vomiting, and vasopressor consumption [7]. Patients were randomized to Groups A and B, receiving variable rates of controlled phenylephrine and norepinephrine, respectively. After the first six minutes following intrathecal injection in Group A, lower systolic blood pressure was observed. There were no statistically significant differences in maternal hypotension between the groups. Group A also demonstrated higher incidences of bradycardia (p=0.001), while hypertension and maternal vasopressor consumption were similar between the groups. In conclusion, as seen in Table 1, dilute norepinephrine (5 µg/mL) was more effective in maintaining blood pressure than phenylephrine (100 µg/mL) in post-spinal anesthesia c-sections, with less bradycardia noted [7]. The purpose of Wang et al. was to compare epinephrine and phenylephrine prevention of post-spinal hypotension after a cesarean section [8]. The 82 patients under observation all received one minute of phenylephrine infusions of 1 μg/kg or epinephrine dose of 0.1 μg/kg for one minute along with intrathecal injection. The patient's vitals and umbilical cord blood gas pre and post-delivery were measured. In addition to this information, the article also took vital note of the occurrence of maternal hypotension, bradycardia, nausea, and vomiting. It was then concluded that blood pressure, heart rate, and cardiac output were all noted to be lower in epinephrine patients than in phenylephrine ones in terms of post-term care. As a result, epinephrine was shown to be more effective and the preferred drug of choice in maintaining blood pressure levels in postpartum women [8].

**Table 1:** Summary of Results

Studies	Study Design, Participant Number (n)	Type of Birth	Type of Anesthesia	Hypotension Drugs Tested and Results	Limitations	Recommendations for Future Studies
Fu et al., 2020 [12]	Randomized Control n = 80	C-section	Spinal prophylactic- methoxamine	The participants randomly received a prophylactic methoxamine infusion with different fixed rates. The study found that maternal hypotension was reduced with increasing methoxamine doses.	Only the first 12 minutes were recorded. Only systolic blood pressure and heart rate were recorded. The study focused on only the healthy part.	More studies on methoxamine should be conducted.
Shang et al., 2021 [11]	Randomized Control n = 33	C-section	Spinal epidural anesthesia- ephedrine requirements with colloid vs crystalloid preloading	This study compared colloid with crystalloid preloading in order to compare hypotension occurrence and subsequent ephedrine use. The group given colloid intervention demonstrated less hypotension than the crystalloid group with a P value of < .00001 furthermore it should be noted that ephedrine dosing .009 and phenylephrine usage .0002 were also lower in the colloid group.	The quality of the evidence might be considered low due to performance and detection bias. There were irregularities in the methodology. The study used ephedrine instead of phenylephrine.	Future studies should focus on phenylephrine instead of ephedrine.
Wang et al., 2020 [8]	Randomized Control n= 82	C- section	Spinal anesthesia	The patients received 1 minute of phenylephrine infusions of 1 μg kg or epinephrine dose of 0.1 μg kg for 1 min synchronously with intrathecal injection. The patient's hemodynamics and umbilical cord blood gasses were measured post-delivery. Epinephrine is more effective at maintaining blood pressure during spinal anesthesia, with a lower decrease in maternal heart rate and cardiac output in relation to phenylephrine.	Researchers did not consider the impact of BMI or maternal age on infusion effectiveness.	Future studies should have a larger sample size.
Goel et al., 2021 [7]	Randomized Double-blind Control n = 200	C-section	Subarachnoid Block (SAB)	Patients were randomized to Groups A and B to receive variable rates of controlled phenylephrine and norepinephrine infusions to maintain systolic blood pressure. Maternal hemodynamics, including hypotension, intraoperative nausea/vomiting, and vasopressor consumption, were measured as well. Lower SBP was observed during the first 6 minutes following intrathecal injection in Group A (receiving phenylephrine). There were no statistically significant differences in maternal hypotension between the groups. Group A also demonstrated higher incidences of bradycardia. Hypertension, IONV, and maternal vasopressor consumption were similar between the groups. Dilute norepinephrine (5 μg​​​​​​​/mL) was more effective in maintaining BP than phenylephrine (10 μg​​​​​​​/mL) in post-spinal anesthesia c-sections, with less bradycardia noted.	Researchers did not measure umbilical blood gasses and cardiac output	More optimized studies are needed to confirm that norepinephrine use is more effective
Yang et al., 2020 [10]	Randomized Control n = 60	C- section	Spinal Epidural Anesthesia	Participants were divided into two groups of 30: standard (Group S, BMI < 30 kg/m2) and obesity group (Group O, BMI > 30 kg/m2). Both groups received combined spinal-epidural anesthesia for c-section. Prophylactic phenylephrine was given to prevent hypotension after a spinal anesthetic was given. The first parturient received an infusion rate pf50 μg/min, which was then increased or decreased by 10 μg/min depending on whether the previous parturient developed hypotension during the study period.	The study contained a small sample size of 56 participants once the exclusion criteria were analyzed.	The dose of phenylephrine for the prevention of hypotension after spinal anesthesia for cesarean section is dependent on maternal BMI. Therefore, a weight-based phenylephrine dose is reasonable
Esen et al., 2021 [13]	Experimental n = 62	C-Section	Spinal	All subjects received lactated Ringer's solution at 10 mL/kg over 15 mins prior to spinal anesthesia. 30 subjects had their legs wrapped with 10-cm Esmarch elastic bandages before spinal anesthesia, and then legs were elevated to 45 degrees. Once the legs were wrapped and elevated, all subjects received 12 mg of hyperbaric 0.5% bupivacaine. Following injection, the subjects' legs were elevated to 20 degrees. Subjects that received leg wrapping and elevation had significantly higher systolic and diastolic blood pressure than the control subjects starting 2 minutes after the intrathecal injection. Additionally, ephedrine dose was significantly higher in the control group compared to the wrapping group (p=0.007). Lastly, vomiting was more frequent in the control group as well (p=0.024).	The researchers did not have a group of subjects that only received leg elevation.	For future studies, a group that undergoes leg elevation without leg wrapping could be utilized to determine if elevation alone is sufficient for increasing blood pressure.
Theodoraki et al., 2021 [9]	Randomized Double-blind Control n = 82	C- section	Spinal-epidural anesthesia	The study compared two infusions: fixed-rate norepinephrine infusion to a fixed-rate phenylephrine infusion. In the norepinephrine group, patients showed lower rates of bradycardic patterns – leading to a lower need for interventions. Phenylephrine recipients showed a higher rate of bradycardia and the need for intervention. There is a possible use of norepinephrine for the management of spinal anesthesia-induced hypotension.	No direct measurement of maternal cardiac output was done, resulting in a favorable effect of norepinephrine.	Include results of elective cesarean sections with morbidity and measure directly maternal cardiac output.

Theodoraki et al. compared two infusions: a fixed-rate norepinephrine infusion to a fixed-rate phenylephrine infusion in a double-blind randomized controlled trial with 82 participants [9]. The parturients were given norepinephrine 4 μg/min or phenylephrine 50 μg/min fixed-rate infusions each starting with the administration of the subarachnoid solution. Maternal hemodynamics were monitored, as well as the incidence of hypotension, and the need for ephedrine or atropine bolus rescue. In the norepinephrine group, patients showed lower rates of bradycardic patterns (p=0.004), leading to a lower need for atropine intervention (p=0.01). Phenylephrine recipients showed a higher rate of bradycardia and the need for intervention. Between the two groups, there was no difference in the number of hypotensive and hypertensive episodes. There is promise seen in the use of the newer drug norepinephrine over phenylephrine for the management of spinal anesthesia-induced hypotension during regional anesthesia in cesarean section due to the lower incidence of bradycardia [9].

Women who are in labor, parturient, will lose a significant amount of blood in the process of delivery. To mitigate that, a prophylactic dose of phenylephrine is given, whose mechanism of action is to bind agonistically to alpha-1 receptors to induce vasoconstriction. In the study demonstrated by Yang et al., the effect of differing maternal BMI values on varying prophylactic doses of phenylephrine after spinal anesthesia was monitored [10]. A standard group (Group S) of women (BMI less than 30 kg/m2) and an obese group (Group O) (BMI greater than 30 kg/m2), were each made up of 30 women who all received spinal-epidural anesthesia for a scheduled c-section. Prophylactic phenylephrine was then given to prevent hypotension. An infusion rate of 50 μg/min was given, which was then increased or decreased by 10 μg/min, depending on whether the previous parturient developed hypotension during the study period. Using the Dixon and Massey method and the isotonic regression method, the results yielded 21.92 μg/min (95% CI, 14.90-28.94 μg/min) for Group S and 42.14 μg/min (95% CI, 24.58-59.70 μg/min) for Group O. As a result, it can be concluded that in cesarean sections, the dose of phenylephrine after spinal-epidural anesthesia is dependent on maternal BMI, thus a weight-based phenylephrine dose is overall reasonable [10].

Colloid and Crystalloid Loading

Shang et al. compared the difference between using colloid solutions and crystalloid solutions to correct hypotension due to neuraxial anesthesia during a cesarean section. The colloid group consisted of various solutions of hydroxyethyl starch while the crystalloid group consisted of various solutions of lactated ringer. The group given a colloid solution had a lower incidence of hypotension compared to the group given a crystalloid solution with a p-value < 0.00001. Within subgroups of the colloid group, the group that received 7-10 mL/kg of 6% hydroxyethyl starch had the lowest incidence of hypotension [11].

Prophylactic Methoxamine Infusions

The prospective double-blinded randomized control study gave prophylactic methoxamine infusions at fixed rates of 1 μg/kg/min (group M1), 2 μg/kg/min (group M2), 3 μg/kg/min (group M3), or 4 μg/kg/min (group M4). Hypotension incidence decreased with increasing methoxamine doses (M1 75%, M2 55%, M3 35%, and M4 10% (P < 0.001)). The only adverse effect that increased, but was not statistically significant, was the incidence of reactive hypertension. Physicians still had to administer boluses of methoxamine during the cesarean section and the randomized control trial (RTC) showed that at lower doses of prophylactic methoxamine infusions, more rescue doses and fluids were necessary (p = 0.0001). The dose-response curve demonstrates an ED50 and ED95 of 2.178 (95% CI 1.564 to 2.680) μg/kg/min and 4.821 (95% CI 3.951 to 7.017) μg/kg/min, respectively [12].

Non-pharmacologic Treatments

Impact of leg wrapping: New research has found that wrapping of the lower extremities, and then elevating them can help to decrease the risk of hypotension in mothers undergoing spinal anesthesia during c-section childbirth. For instance, Esen et al. used 10-cm Esmarch elastic bandages to wrap from the ankle to the mid-thigh bilaterally and then elevated the subjects’ legs to 45 degrees before the administration of the spinal block [13]. Following the administration of 12 mg of hyperbaric 0.5% bupivacaine, the subjects’ legs were then lowered to 20 degrees, and blood pressure was taken [13]. The results of this experiment showed that after three minutes of intrathecal injection and for 30 minutes, the subjects that had their legs wrapped had a significantly higher systolic and diastolic blood pressure and ephedrine dose than the control (p=0.007) [13].

Discussion

The study focused on the review of different modalities of treatment, both pharmacologic and nonpharmacologic, to reduce the rate of neuraxial anesthesia-induced maternal hypotension during cesarean section delivery. This review found that agents such as epinephrine and norepinephrine, rather than the widely used phenylephrine, were more efficacious in maintaining maternal blood pressure and preserving other hemodynamic factors. Epinephrine and norepinephrine are more efficient and have a better therapeutic impact during spinal anesthesia. Per the research, the main benefits of using epinephrine or norepinephrine in place of phenylephrine include decreased bradycardia, nausea, vomiting, and most significantly, decreased incidence of hypotension. Further research is needed to determine the single best treatment modality, but the overall goal of this research is to find which modality can better address anesthesia-induced hypotension during labor and delivery via c-section, and how we can safely treat mothers during this time.

Specifically in post-spinal anesthesia c-sections, dilute norepinephrine (5 µg/mL) was noted to be more advantageous than phenylephrine [7]. Furthermore, norepinephrine was also the drug of choice in terms of treating symptoms of hypotension and bradycardia in postpartum women [8]. Although there are several theories as to why this is the case, the leading theory appears to be connected to the reduced arterial compliance that norepinephrine causes compared to phenylephrine. Phenylephrine is a pure alpha-1 adrenergic agonist that leads to vasoconstriction, whereas norepinephrine has both alpha and beta-adrenergic effects which acts as a vasoconstrictor and inotrope. Norepinephrine can increase cardiac output and lead to cardiovascular coupling better than phenylephrine; as a result, it is being proposed as the first-line vasopressor during post-anesthetic care [14].

Even though many studies are comparing the various treatment modalities of neuraxial anesthesia, specifically spinal anesthesia-induced hypotension, there is a lack of research looking more into why some modalities such as epinephrine and norepinephrine are working better. Furthermore, there should be more emphasis on projects to delve further into whether there are factors as to why epinephrine and norepinephrine are more effective in postpartum mothers specifically. Numerous pathophysiological changes occur in pregnancy, especially related to the heart. Arterial compliance specifically increases during pregnancy; there is a “steady and pulsatile decrease in afterload” [15]. As discussed above, since epinephrine and norepinephrine affect arterial compliance and afterload, that might be a hypothesis indicated as to why those drugs are proving to be more effective.

Currently, it is standard practice to combine crystalloids and a continuous infusion of phenylephrine immediately after intrathecal anesthetic injection to manage hypotension during c-sections [4]. Various articles discussed above demonstrate that colloid solution also significantly lowers the incidence of hypotension in postpartum mothers, specifically 7-10 mL/kg of 6% hydroxyethyl starch. Many incidences of hypotension, specifically in childbirth settings are related to hypovolemia as well. Crystalloids dilute protein plasma content which reduces plasma oncotic pressure and overall helps to address the incidence of hypotension [16]. Colloids, due to their larger molecular size in comparison to crystalloids, are more effective in fluid resuscitation, which can be helpful postpartum. Colloid and crystalloid solutions should be explored further in postpartum hypotension settings especially if the patient requires fluid resuscitation as well. This can be an effective and monetarily advantageous solution to both hypotension and hypovolemia, which are common in the post-spinal anesthetic population. 

Another modality to avoid maternal hypotension is methoxamine infusion. Methoxamine infusion is unique in that it is a prophylactic solution; it is given before the mother begins the birthing process to avoid potential complications. Methoxamine is a pure alpha-1 receptor agonist and strictly causes peripheral vasoconstriction [17]. Studies show that doses of 1-4 μg/kg/min can be used to avoid the complication of maternal hypotension [12]. While rebound hypertension is a potential side effect of prophylactic methoxamine, the benefits of using it outweigh the risks. By using methoxamine infusion as a method of preventing maternal hypotension from the start of the laboring process, healthcare systems are able to preserve resources that would have been used to treat the hypotension. More importantly, methoxamine infusions allow for the safety of the mother and fetus during the laboring process. 

The technique of leg wrapping with an elevation of the lower extremities demonstrated statistically significant increases in blood pressure compared to the control group [13]. Given the simplicity of this modality, as well as the availability and lower cost compared to pharmaceutical agents, future research examining this method has a lot of potential. Researchers in the future could examine combining pharmacologic intervention with modalities such as leg wrapping to optimize treatment and limit adverse effects in the mother or fetus and potentially increase the therapeutic effects of both treatments. 

This review exhibits many different modalities to prevent neuraxial anesthesia-induced hypotension during labor and delivery. Clinical practices should use the outlined conventions when treating this population of patients to decrease the incidence of complication, morbidity, and mortality in labor and delivery. In future research on this topic, it is important to consider and explore both pharmacologic and non-pharmacologic interventions. 

Limitations

The inclusion criteria indicated that articles had to be peer-reviewed, written in English, and conducted on or after January 1st, 2018. Based on the inclusion criteria the scoping review was limited to analyzing six studies. This limited the sample size and methodology of treatment that was assessed for each modality. Additionally, limitations specific for each individual article have been outlined in Table 1. The study criteria also limited our participants to women under 35 years old with no pre-existing comorbidities. The most significant comorbidities that were excluded included gestational diabetes, hypertension, and advanced gestational age, therefore the results did not take into consideration a large population of pregnant women. Lastly, to standardize the method of childbirth across the study, c-section births, and only nulligravida and primigravida mothers were included to decrease complications that may be associated with being multigravida rather than the anesthesia.

Future research implications

Future research directions should include comparing epinephrine, norepinephrine, and phenylephrine to ascertain which medication is most efficacious in treating neuraxial anesthesia-induced hypotension in labor and delivery. The exploration of both pharmacological and nonpharmacological interventions should be an imperative part of advancing research in this field. To facilitate this investigation, a three-arm study should be conducted, encompassing the current standard of care, the addition of an adjuvant, and a nonpharmacological approach such as the use of compression stockings or leg wrapping. This type of randomized control trial would allow analysis between pharmacological and nonpharmacological interventions, which is a crucial consideration especially when pharmacological contraindications may be present. Additionally, researchers could also combine pharmacologic intervention with leg wrapping to optimize treatment and potentially increase the therapeutic outcomes. 

Furthermore, future research should focus on examining pregnant women with comorbidities such as diabetes, obesity, and advanced maternal age. Investigating these demographics can provide valuable insight into challenges and potential interventions to improve outcomes for this population. By focusing on medical therapy and interventions, researchers can contribute to a more comprehensive understanding of the health requirements and outcomes for all pregnant women. This approach has the potential to lead to improved healthcare strategies tailored to the specific needs of pregnant women, ultimately benefiting both current and future generations.

## Conclusions

Maternal hypotension remains a persistent concern following the administration of neuraxial anesthesia, both of which are heavily used in the United States. Society must focus on minimizing the adverse side effects of these mechanisms. Hypotension has a range of negative adverse effects that can affect not only the mother long term but also the newborn. By proactively managing maternal blood pressure, healthcare providers can effectively prevent these complications, with hopes of decreasing maternal stay in the hospital and reducing the cost of care for both the hospital and the patient.

Based on the findings of this scoping review, it is evident the most effective method of reducing post-neuraxial anesthesia hypotension is pharmacological interventions. While epinephrine, norepinephrine, and phenylephrine can all demonstrate the capacity to decrease maternal hypotension, epinephrine stands out due to its lower incidence of maternal bradycardia. In contrast, when considering non-pharmacological interventions, colloid preloading surpasses crystalloid preloading by not only decreasing the necessary amount of ephedrine but is also the most effective at decreasing the incidence of maternal hypotension, nausea, and vomiting in comparison. Overall, colloid preloading emerges as one of the most efficient and cost-effective roles in reducing the adverse effects of anesthesia. However, other non-pharmacological interventions including leg wrapping and leg elevation can also be utilized to decrease the incidence of hypotension. 

With this valuable information, physicians can make more educated and informed decisions on how to treat maternal hypotension effectively. In the future, various groups such as the American College of Obstetricians and Gynecologists and the American Society of Anesthesiologists can collaborate to utilize this review as a resource to develop comprehensive guidelines for the treatment of neuraxial analgesia induced maternal hypotension where guidelines and protocols are not already in place or to improve upon guidelines or protocols that are already in place. This can be done while taking into account various factors such as pre-existing medical conditions and maternal age.
